# David D. Allen to lead University of Health Sciences and Pharmacy in St. Louis

**DOI:** 10.1093/ajhp/zxab172

**Published:** 2021-05-06

**Authors:** Kate Traynor

In a year marked by change, David D. Allen is making a notable move to become the fifth president of the University of Health Sciences and Pharmacy (UHSP) in St. Louis, MO.

**Figure:**
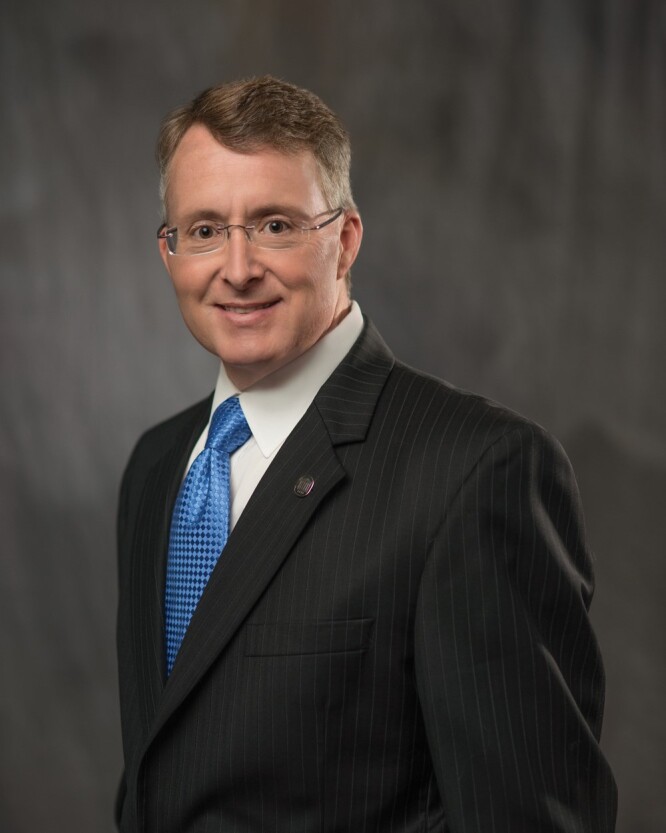
David D. Allen

Allen officially begins his new position July 1. He succeeds retiring UHSP President John A. Pieper.

“It’s a fantastic opportunity,” Allen said. He said his goals as president are to support the success of students, staff, and the university as a whole.

UHSP Board of Trustees Chair Jo Anne Levy said Allen arrives at a pivotal time for the university.

“Dr Allen has the skills, experience, and passion to lead UHSP as we build on our history and look forward to many exciting opportunities,” Levy said. She said Allen’s leadership will facilitate sustained excellence as the university expands while providing “an enriching interprofessional community where students can grow personally and professionally as skilled, empathetic professionals and providers who will make an impact in their communities.”

Allen has served since 2012 as dean of the University of Mississippi School of Pharmacy, professor of biomolecular sciences, and executive director and research professor of the university’s Research Institute of Pharmaceutical Sciences. Previously, he was founding dean of pharmacy and professor of pharmaceutical sciences at Northeast Ohio Medical University College of Pharmacy in Rootstown.

Allen said he’s eager to step into his larger academic role and take on the challenges that are affecting universities.

“It’s a transformational time for higher education. We’re in the middle of the COVID-19 pandemic, and we’ve all redone how we do business from a year ago, pivoting on a dime to become much more virtual,” Allen said.

He said big questions in higher education include what the post–COVID-19 new normal will be and how to recruit talented students at a time when they and their families are increasingly concerned about the cost and value of their academic investment.

“I think pharmacy school and higher education in general have to be very nimble and flexible to be able to respond to the new students,” Allen said. “We’ve got to be very thoughtful about creating a new path forward that’s going to be attractive to students.”

He emphasized that the new path must emphasize equity, diversity, and inclusion (EDI)—a personal passion for Allen and a happy legacy he leaves behind as outgoing dean of the University of Mississippi School of Pharmacy.

Allen explained that the pharmacy school partnered with the American Association of Colleges of Pharmacy (AACP) to develop the Equity, Diversity and Inclusion Institute. The inaugural institute was held virtually in January, with the goal of helping attendees develop an EDI action plan for their organization.

“The more that we can engage in that realm, the more effective we can be as a profession at taking care of our patients,” said Allen, a longtime member and past president of AACP. “It’s something I’ve been deeply committed to as a pharmacist and as a person for a long, long time.”

Allen received a bachelor’s degree in pharmacy and a PhD in pharmaceutical sciences from the University of Kentucky. He then worked as a neuroscience researcher and Intramural Research Training Award Fellow at the National Institute on Aging within the National Institutes of Health (NIH) in Bethesda, MD.

Over the course of his career, Allen has been a principal investigator on more than 35 NIH-funded research projects. He said he considers himself a pharmacist first, but his deep roots in research influence his views on the profession and its future.

“Pharmacy is a point of convergence, I think, between discovery and action; between research and patient care,” he said. “I think that it’s extremely important for us to think, going forward, that we are both scientific and patient focused. And we function as a profession in both realms of that overall endeavor.”

Allen entered academia when he joined the Texas Tech University Health Sciences Center School of Pharmacy in Amarillo, serving as a founding faculty member and associate dean of curricular affairs. He is a past president of the Texas Society of Health-System Pharmacists (TSHP), and he credited the ASHP affiliate with teaching him leadership skills that have helped him grow professionally.

“Before I was president, the very first leadership opportunity I ever had was as a member of the board for TSHP,” Allen recalled. “They literally had a leadership development program for the board members. So while I’ve had many other forays into leadership development, my first significant one was . . . as a member of the TSHP board of directors.”

Allen joined ASHP in 1999 while on the faculty at Texas Tech. He has participated in many ASHP councils and served in the House of Delegates. He said professional organizations like ASHP, by representing pharmacists as patient-care providers, “help to raise the profile of the pharmacy profession and highlight the diverse roles that pharmacists can bring, not only to patients specifically but to healthcare in general.”

ASHP named Allen a Fellow in 2004. He is also a Fellow of the National Academies of Practice and the American Pharmacists Association.

